# Supplementary material for: Quantified Vascular Calcification at the Dialysis Access Site: Correlations with the Coronary Artery Calcium Score and Survival Analysis of Access and Cardiovascular Outcomes
Source: J Clin Med. 2020 May 21;9(5):1558. doi: 10.3390/jcm9051558 (PMC7290563; doi:10.3390/jcm9051558)

Supplementary Material

Supplementary Figure 1. Flow chart of the subjects

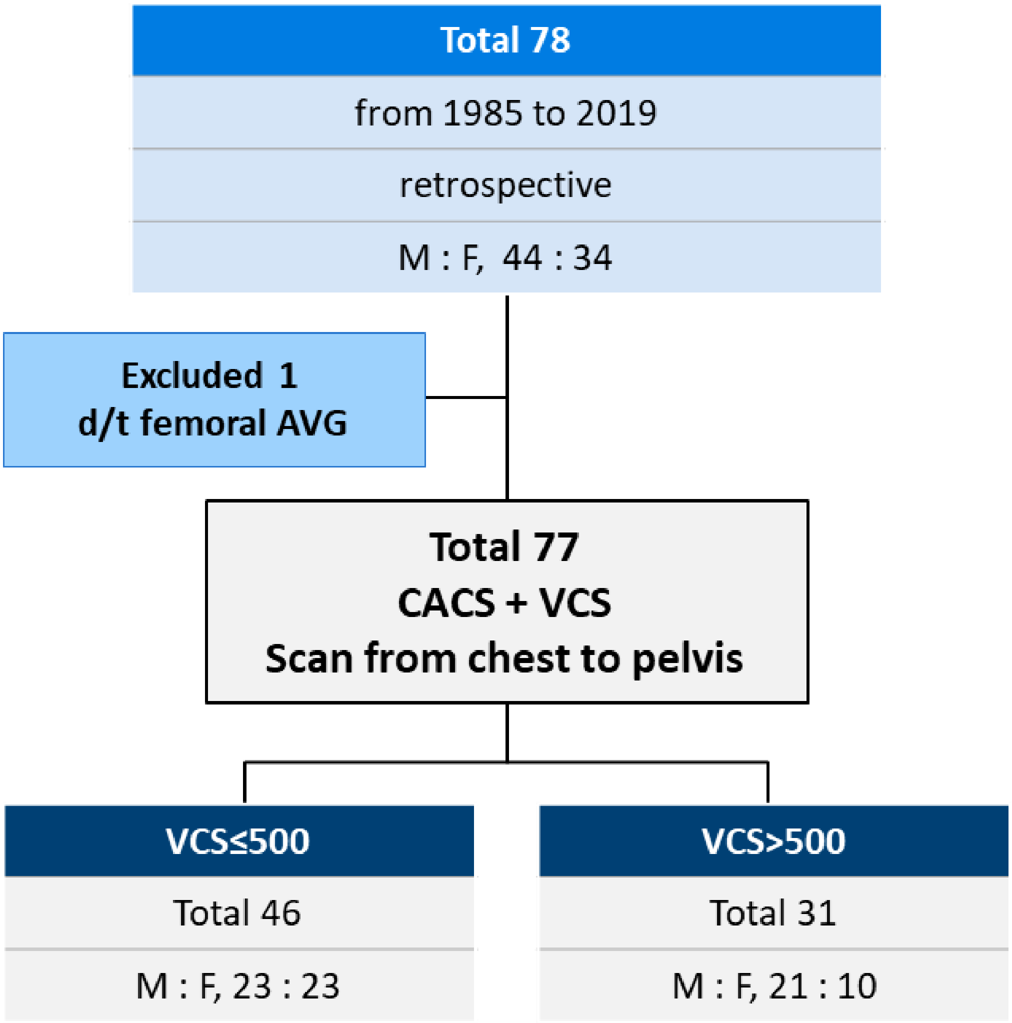

Supplementary Figure 2. The distribution of magnesium in our study

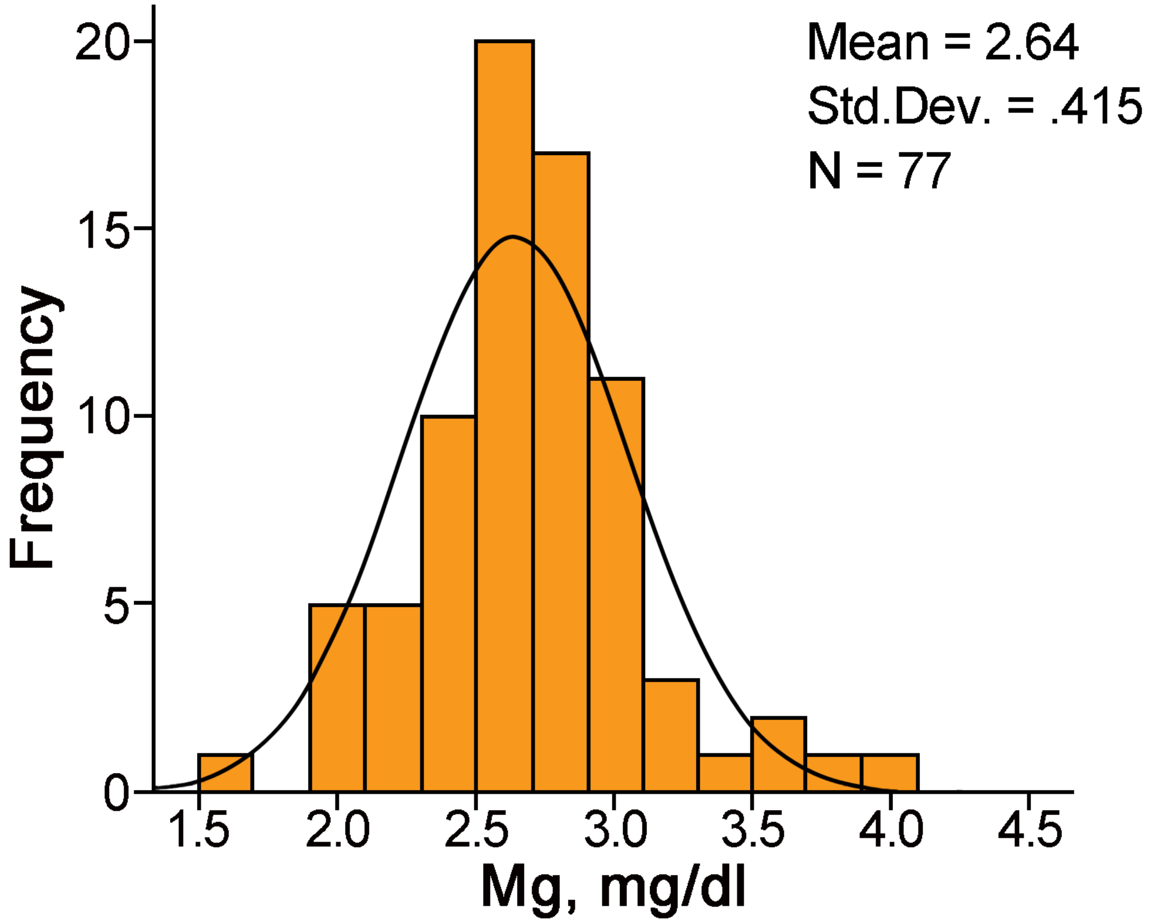

Supplement: Supplementary file 1 [file jcm-09-01558-s001.pdf]
